# The Activity of *Tc*CYS4 Modified by Variations in pH and Temperature Can Affect Symptoms of Witches’ Broom Disease of Cocoa, Caused by the Fungus *Moniliophthora perniciosa*

**DOI:** 10.1371/journal.pone.0121519

**Published:** 2015-04-01

**Authors:** Ana Camila Oliveira Freitas, Cristiane Ferreira Souza, Paulo Sérgio Monzani, Wanius Garcia, Alex Alan Furtado de Almeida, Marcio Gilberto Cardoso Costa, Carlos Priminho Pirovani

**Affiliations:** 1 Centro de Biotecnologia e Genética, Universidade Estadual de Santa Cruz (UESC), Ilhéus, Bahia, Brazil; 2 Centro de Biologia e Ciêncais da Saúde, Universidade do Norte do Paraná, Londrina, Paraná, Brazil; 3 Centro de Ciências Naturais e Humanas (CCNH), Universidade Federal do ABC (UFABC), Santo André, Sao Paulo, Brazil; Universidade Federal do Rio Grande do Sul, BRAZIL

## Abstract

The phytocystatins regulate various physiological processes in plants, including responses to biotic and abiotic stresses, mainly because they act as inhibitors of cysteine proteases. In this study, we have analyzed four cystatins from *Theobroma cacao* L. previously identified in ESTs libraries of the interaction with the fungus *Moniliophthora perniciosa* and named *Tc*CYS1, *Tc*CYS2, *Tc*CYS3 and *Tc*CYS4. The recombinant cystatins were purified and subjected to the heat treatment, at different temperatures, and their thermostabilities were monitored using their ability to inhibit papain protease. *Tc*CYS1 was sensitive to temperatures above 50°C, while *Tc*CYS2, *Tc*CYS3, and *Tc*CYS4 were thermostable. *Tc*CYS4 presented a decrease of inhibitory activity when it was treated at temperatures between 60 and 70°C, with the greater decrease occurring at 65°C. Analyses by native gel electrophoresis and size-exclusion chromatography showed that *Tc*CYS4 forms oligomers at temperatures between 60 and 70°C, condition where reduction of inhibitory activity was observed. *Tc*CYS4 oligomers remain stable for up to 20 days after heat treatment and are undone after treatment at 80°C. *Tc*CYS4 presented approximately 90% of inhibitory activity at pH values between 5 and 9. This protein treated at temperatures above 45°C and pH 5 presented reduced inhibitory activity against papain, suggesting that the pH 5 enhances the formation of *Tc*CYS4 oligomers. A variation in the titratable acidity was observed in tissues of *T*. *cacao* during the symptoms of witches’ broom disease. Our findings suggest that the oligomerization of *Tc*CYS4, favored by variations in pH, is an endergonic process. We speculate that this process can be involved in the development of the symptoms of witches’ broom disease in cocoa.

## Introduction

The fermented seeds of cocoa (*Theobroma cacao* L), also known as beans, are considered a commodity, since they serve as raw material for the chocolate industry. Fungal diseases promote large losses in the production of beans. The witche’s broom disease (WBD) caused by the fungus *Moniliophthora perniciosa* is an important disease in cocoa producing areas of Central and South America [1,2]. The fungus *Moniliophthora perniciosa* (formerly *Crinipellis perniciosa*) [3] is a hemibiotrophic basidiomycete. The biotrophic or parasitic phase is characterized by the presence of monocariotic mycelium in the intercellular space, causing the loss of apical dominance, hyperplasia, hypertrophy, and proliferation of axillary branches called green brooms [4]. In the necrotrophic or saprophytic phase, the hyphae are slender, dicariotic, and present clamp connections between the septa. At this stage, the plant’s infected tissue is necrotic and dead, forming dry brooms [4–8].

During the “molecular battle”, the fungus can overcome the initial defense barriers of *T*. *cacao*, and, as a defense strategy, the release of reactive oxygen species (ROS) occurs due to an oxidative burst that does not quite characterize a hypersensitivity reaction (HR) [9,10]. A series of changes in the antioxidant system of *M*. *perniciosa*-susceptible *T*. *cacao* genotypes generate a breakdown in the mechanism of protection of the host that leads to programmed cell death (PCD) and death of the plant’s tissues. At this stage, the fungus benefits from the increased availability of nutrients and changes from biotrophic to necrotrophic phase [5].

Homologous of the four classes of proteases (serine, cysteine, metallo, and aspartic proteases) related to PCD and defense of *T*. *cacao* were identified in ESTs libraries of the interaction between cacao and *M*. *perniciosa* [11]. A papain-like cysteine protease (PLCP) presented greater accumulation in the final stages of the parasitic phase of the disease [12]. In addition, four protease inhibitors from *T*. *cacao* have been identified in ESTs libraries and named *Tc*CYS1 (KM361432), *Tc*CYS2 (KM361433), *Tc*CYS3 (KM361434), and *Tc*CYS4 (KM361435) [13]. These proteins have a molecular mass of 21.5, 14, 11.6 and 22.8 kDa, respectively. They were expressed in *Escherichia coli* and the four recombinant proteins affected the growth of *M*. *perniciosa* [13]. *Tc*CYS4 presented greater accumulation in young tissues and during the parasitic phase of the disease, with reduction of accumulation at the end of this phase [13].

The phytocystatins (PhyCys) are inhibitors of specific cysteine proteases of plants that present three sites involved in the interaction with papain-like proteases: one or two glycine residues in the N-terminal part of the protein, the reactive site QxVxG, and one tryptophan located after the active site [14]. Furthermore, the carboxy-extended region has the ability to inhibit cysteine proteases and also legumain-like [15]. The PhyCys participate in many cellular processes, such as: programmed cell death (PCD), based on its capacity to modulate active cysteine proteases [15]; inhibition of proteases from the digestive tract of insects and nematodes [16, 17]; control of the activity of cysteine proteases that are involved in recycling processes of proteins during senescence [18]; and also in the activation of the protection of the metabolic pathways in conditions of abiotic stress [19]. The cystatins are monomeric proteins, with the exception of human cystatin F, which is found as inactive dimers linked by disulfide bond [20]. The dimeric form with disulfide bonds reduces the activity of cystatins. The formation of non-covalent homodimers was observed in cystatin C subjected to stressing conditions, at temperatures between 60 and 80°C and low pH [21]. The oryzacystatin-II from rice forms homodimers when stored at 4°C; however, the dimers are converted to monomers when subjected to 65°C [22].

In this study, we evaluated the effects of heat treatments on the recombinant *Tc*CYS1, *Tc*CYS2, *Tc*CYS3, and *Tc*CYS4 cystatins from *T*. *cacao*, in relation to the cysteine protease inhibition activity and the formation of dimers. *Tc*CYS4 forms stable homodimers when treated at 65°C *in vitro* and dimerization is favored at pH 5, as suggested by the activity pattern observed for the protein subjected to heat treatment at this pH. A variation of acidity in cocoa tissues during the interaction with *M*. *perniciosa* was detected, with higher acidity in the late biotrophic phase. We suggest that oligomerization of endogenous *Tc*CYS4 protein may be associated with the development of the symptoms of WBD in cocoa.

## Material and Methods

### Expression and purification of recombinant cystatins from *Theobroma cacao*

The cDNA corresponding to the *Tc*CYS1, *Tc*CYS2, *Tc*CYS3, and *Tc*CYS4, identified from ESTs libraries of the interaction between *Theobroma cacao* and *Moniliophthora perniciosa* [11], were subcloned into pET28a(+) (Novagen) bacterial expression vector according to previously described [13]. The recombinant cystatins were successfully expressed in *Escherichia coli* in soluble form and purified as previously described by Pirovani et al. [13]. The purity of the recombinants proteins were checked by 15% SDS-PAGE, subsequently dialyzed against the working buffer (variable depending on the assay), and the concentrations of the recombinant proteins were determined employing the Bradford method [23]. The final purified proteins were concentrated at 1 mg.mL^-1^, stored at 8°C, and kept on ice before use.

### Papain Inhibitory assays

The quantitative analyses of the papain inhibitory activity of recombinant cystatins from cocoa were performed as previously described [13]. The protease used was papain from papaia latex (P4762, Sigma) and the substrate was Nα-Benzoyl-DL-arginine 4-nitroanilide hydrochloride (B4875, Sigma). Each assay was modified according to the experiments: (i) for inhibitory activity characterization, after heat treatment, the aliquots of the cystatins at 20 μM were submitted at different temperatures during 10 min in activity buffer (100 mM phosphate buffer at pH 6, 2 mM EDTA, and 10 mM β-mercaptoethanol); (ii) for *Tc*CYS4 dimers thermostability assay, six aliquots of the protein were incubated at 65°C during 10 min and stored at 8°C over twenty days and evaluated; (iii) for dimers to monomers conversion assay, the aliquots of the protein *Tc*CYS4-His-tag and *Tc*CYS4 (His-tag was removed by prior treatment with thrombin) were incubated during 10 min at 65°C and 80°C; (iv) for the analysis of the influence of time of exposure to the temperature on the formation of dimers, the aliquots of *Tc*CYS4 were treated from 0 to 10 minutes at 65°C; (v) for the assessment of the influence of pH on the *Tc*CYS4 inhibitory activity, the aliquots were treated at different pH values using 100 mM phosphate buffer and 250 mM glycine, and incubated during 10 min at 37°C; (vi) for the analysis of the influence of pH on the secondary structures, aliquots of *Tc*CYS4 were incubated during 1 hour at 25°C in phosphate buffer or 250 mM glycine at pH 4, 5, 6, 7, and 8, and then the aliquots were heat-treated for 10 min at different temperatures. To calculate the inhibition percentage (i %), the following relationship was used: i % = [(F—I)/ C] X 100%; in which F corresponds to final OD_410nm_ of the reaction with the inhibitor; I corresponds to the initial OD_410nm_; and C is the mean of the final OD_410nm_ of the control reaction (no inhibitor). In order to calculate the percentage of residual activity of papain, the final OD_410nm_ of the reaction in the absence of *Tc*CYS4 as control reaction (C) was used.

### Determination of the oligomeric state of recombinant *Tc*CYS4 by native gel electrophoresis and size-exclusion chromatography (SEC)

Aliquots of the protein *Tc*CYS4 at 20 μM treated at different temperatures for 10 min received non-denaturing sample buffer (20% v/v Glycerol, 200 mM Tris-HCl pH 6.8 and 0.05% w/v bromophenol blue) and were analyzed on 15% native gel electrophoresis, according to [24], using an electrophoresis chamber model SE260 (Omniphor). The gels were made in triplicate and the three images of the native gels were scanned in an ImageScanner (Amersham Biosciences) and the bands were quantified using the ImageMaster 2D-Plantinum 7.0 Software (GEHealthCare). Aliquots of the protein *Tc*CYS4 at 20 μM in 100 mM phosphate buffer at pH 6 containing 150 mM NaCl were treated at different temperatures during 10 min, and subsequently analyzed by size-exclusion chromatography (SEC), using a Superdex 75 5/150 column (GEHealthcare) in HPLC ÄKTAPurifier (GEHealthcare). The molecular weight marker LMW (GEHealthcare) in 100 mM phosphate buffer at pH 6 and containing 150 mM NaCl was used as protein standards. This marker contains bovine albumin (67 kDa), ovalbumin (43 kDa), chymotrypsinogen A (25 kDa), and ribonuclease A (13.7 kDa). The samples were analyzed throught the retention volume, in the column previously equilibrated with 100 mM phosphate buffer at pH 6 containing 150 mM NaCl, under a flow of 150 μL.min^-1^ and monitored at 280 nm.

### Circular dichroism spectroscopy

The circular dichroism (CD) spectroscopy measurements were carried out in spectropolarimeter J-815 (Jasco) equipped with a *Peltier* PTC-423S/15 temperature control unit. CD measurements were performed using a 1 mm path-length quartz cuvette, scan rate of 50 nm.minute^-1^, and interval of data collection of 0.5 nm. The CD spectra of *Tc*CYS3 and *Tc*CYS4 were recorded over the wavelength range from 180 to 250 nm and were determined as an average of 16 scans. The protein was used at a concentration of 1 mg.mL^-1^ in 10 mM sodium phosphate buffer at pH 6. Aliquots treated at 26°C, 65°C, and 92°C during 5 min were analyzed using the Spectra Measurement Software (Jasco).

The influence of pH associated with the heat treatment on *Tc*CYS4 inhibitory activity was assessed for samples obtained in four conditions: (i) maintained at 8°C in 250 mM sodium phosphate buffer at pH 6; (ii) treated at 65°C during 10 min in 250 mM sodium phosphate buffer at pH 6; (iii) treated in 250 mM sodium phosphate buffer at pH 5 during 30 min at 25°C; (iv) and treated with 250 mM sodium phosphate buffer at pH 5 during 30 min at 25°C and then treated during 10 min at 65°C. The scans were performed in the spectra from 200 to 250 nm at the temperatures of 26°C and 65°C depending on the treatment, with 5 min of incubation of the sample before starting the readings. Three consecutive measurements were performed and the mean of the three spectra was utilized.

Thermal denaturation (unfolding) of *Tc*CYS4 (at 20 μM), without and with previous treatment at 65°C during 10 min, was characterized by measuring the ellipticity changes at 218 nm induced by a temperature increase from 26 to 92°C. Reversibility of *Tc*CYS4 denatured (refolding) was assessed acquiring the CD spectra of the sample at the same initial condition after heating to 92°C.

### Titratable acidity in cocoa leaf tissue

The leaf material was collected from “Common Cocoa” (*Theobroma cacao* L. var. “Parazinho”), which is susceptible to the fungus *M*. *perniciosa*, in a cultivated area in Ilhéus, Bahia, Brazil. The third fully expanded leaf from the apex of plagiotropic branches displaying different stages of infection by *M*. *perniciosa* was collected according to [5,11,25], as following: leaves from healthy branches; stage I—leaves from branches with early symptoms of parasitic phase; stage II—leaves from branches with well-established symptoms of parasitic phase; stage III—leaves from branches in transition between the parasitic and saprophytic phase, with initial necrosis. After the collects, the plant material was immediately stored in liquid N_2_ and then lyophilized.

The mass of approximately 0.2 g of tissue between the 2nd and the 3rd secondary vein of the leaf was used, with five replicates for each stage. After weighing, 25 mL of distilled water were added to the dried mass and boiled for 15 min. Then, the plant material was removed from the container and the volume was completed to 50 mL with distilled water. The samples were cooled at room temperature (~25°C) and added two drops of 1% phenolphthalein solution. After, they were submitted to titration with sodium hydroxide at 10 mM, being standardized with sodium biphthalate at 10 mM. The titratable acidity was expressed in μmol of H^+^.g of lyophilized mass^-1^.

## Results

### Heat stability of recombinant cystatins from *T*. *cacao*

**Fig 1:**
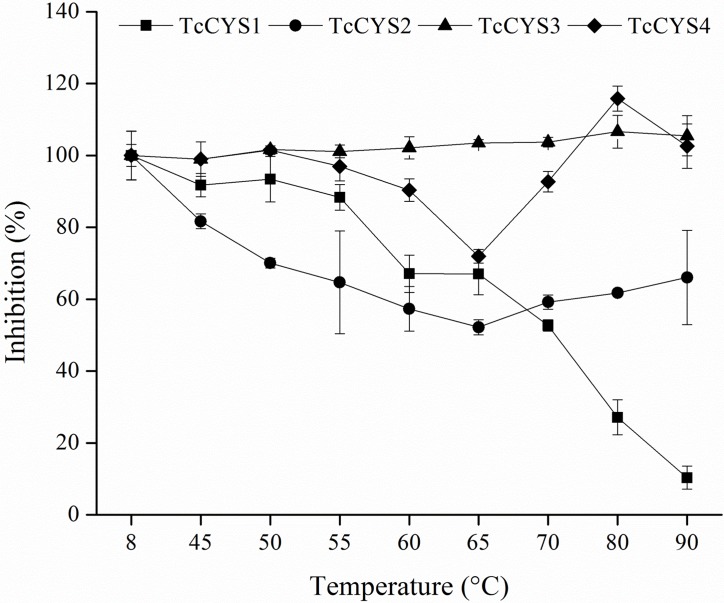
Inhibitory profile of the four *T*. *cacao* cystatins after heat treatment. *Tc*CYS1 protein(■) is heat unstable, and the *Tc*CYS3 protein(▲) is heat stable. *Tc*CYS2 proteins(●) and *Tc*CYS4 (♦) also presented heat-stable behavior; however, they present reduction of inhibitory potential after treatments in the temperature range between 60 and 70°C. The vertical bars correspond to the standard deviations of the mean (n = 3).

The four recombinant cystatins from cocoa were treated at different temperatures for 10 min and then submitted to the papain inhibitory activity assay (Fig. 1). *Tc*CYS1 gradually lost its inhibitory capacity with the increase in temperature after 50°C. In the treatment at 70°C, it showed 50% of inhibitory activity, and at 90°C it completely lost its activity. This protein presented precipitation caused by the increase in temperature (data not shown).

*Tc*CYS2 showed reduced inhibitory activity when pretreated at temperatures of 45 to 65°C, presenting around of 52% of the activity at this temperature. The protein activity has been slightly increased from 70 to 80°C, reaching about 65% of inhibitory activity at 90°C. This protein also precipitated in the treatments above 45°C (data not shown), but in an amount less significant than in *Tc*CYS1.

*Tc*CYS3 did not show alterations in the inhibitory activity in all the heat treatments analyzed. This protein maintained the inhibition rate at approximately 100% after the treatments for 10 min at temperatures up to 90°C. The curves for unfolding and refolding by CD of *Tc*CYS3 were superimposed (S1 Fig.).

*Tc*CYS4 reduced the inhibitory capacity when the temperature was increased to 65°C, showing 70% of activity at this temperature. However, it was evidenced a gradual increase in the activity when the temperature was risen from 70 to 90°C, with more than 100% of activity when treated at 80°C (Fig. 1).

### *Tc*CYS4 dimers

**Fig 2:**
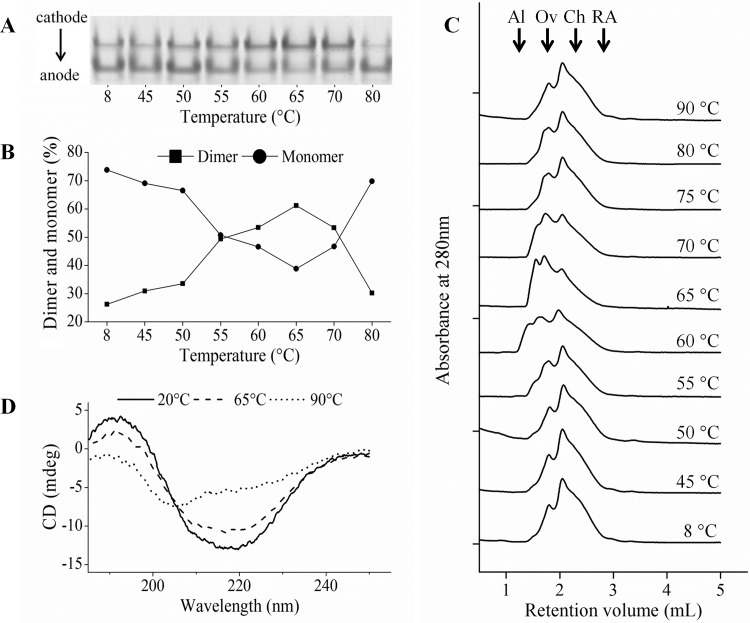
Heat treatment at 65°C induces the formation of recombinant *Tc*CYS4 dimers. **A**, native gel of *Tc*CYS4 incubated at temperatures ranging between 45 and 80°C for 10 minutes. **B**, percentages of dimers and monomers of *Tc*CYS4 obtained by densitometry of the gels of A in triplicate using the software Image2D Platinum 7.0. **C**, size exclusion chromatography of *Tc*CYS4 after heat treatment. The retention volumes for the standards are indicated as Al, BSA with 66.5 kDa; Ov, ovalbumin with 45 kDa; Ch, Chymotrypsinogen A with 25 kDa; AR, ribonuclease A with 13.8 kDa. The size expected for monomers, dimers ande trimers for His-tagged protein is approximately 24, 48 and 72 kDa, respectively. **D**, Spectral profiles by circular dichroism at the wavelengths from 190 to 250 nm of the *Tc*CYS4 treated at 20°C (solid line), 65°C (dashed line), and 90°C (dotted line) showing that the protein undergoes minor alterations at 65°C, and, when analyzed at 90°C, it undergoes loss of structure when compared with the protein at 20°C.

*Tc*CYS4 presented a peak of inhibitory activity loss in the treatment at 65°C. Thus, its oligomeric state was investigated by native gel electrophoresis (Fig. 2A and B) and size-exclusion chromatography (Fig. 2C).

In the treatments at 60, 65, and 70°C, the protein showed intensification of the bands near to the cathode and, proportionately, a reduction in the intensity of the bands near to the anode (Fig. 2A and B). The analysis of aliquots of the protein submitted to heat treatment through size-exclusion chromatography showed the occurrence of monomers and dimers in all the heat treatments evaluated, as well as the occurence of trimers in the range from 55 to 75°C (Fig. 2C). In addition, the increased formation of oligomers and reduction of monomers were observed in the range from 60 to 70°C (Fig. 2C). CD spectroscopy showed little alteration in the regular secondary structures of the protein when it was compared with the treatments at 20 and 65°C. The treatment at 90°C altered the CD spectrum of the protein *Tc*CYS4 (Fig. 2D).

### Formation and stability of the oligomers

**Fig 3:**
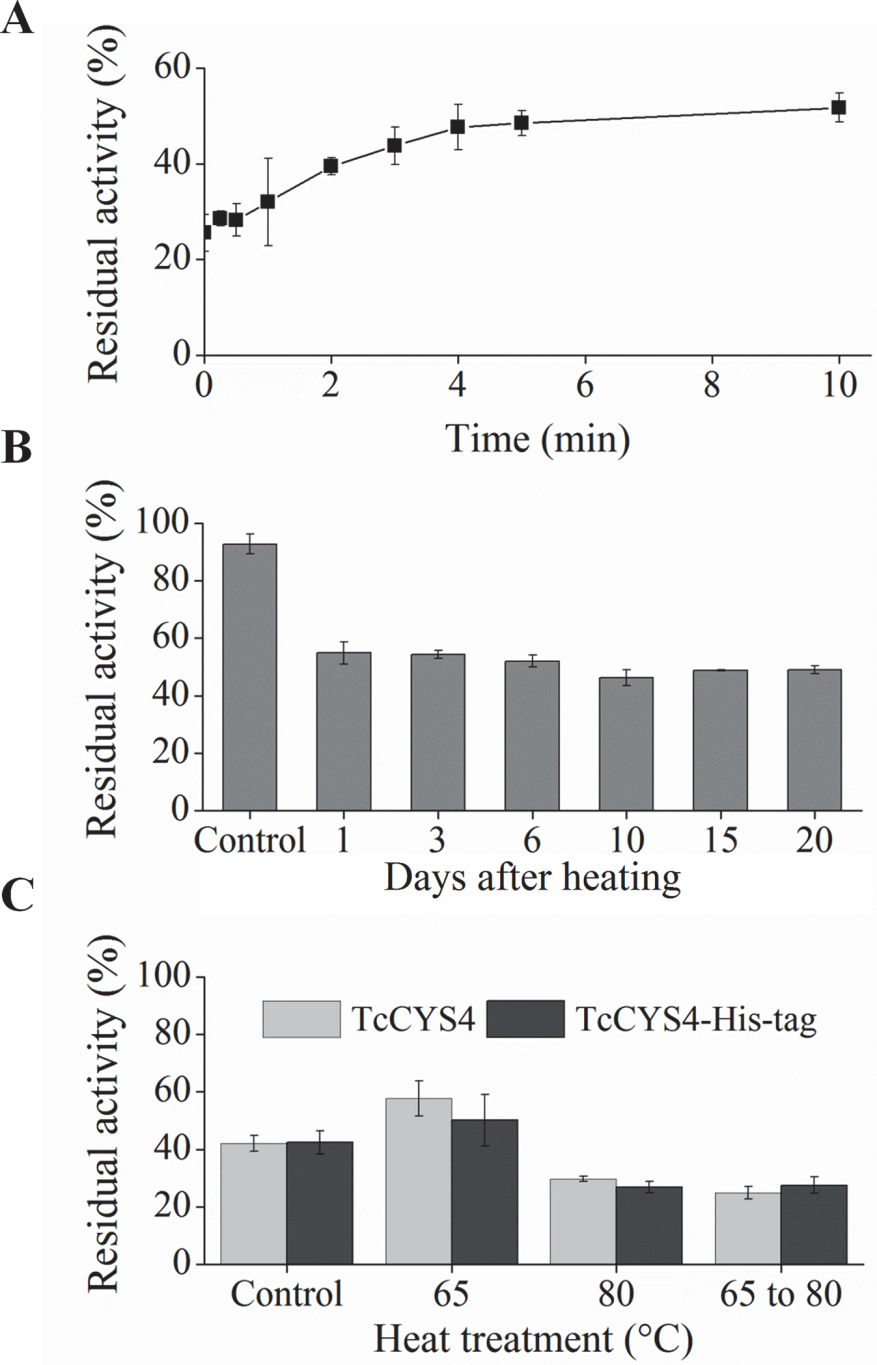
The*Tc*CYS4 dimers are stable after storage at 8°C. A, residual activity of papain in reactions containing aliquots of the protein treated at 0, 0.4, 0.5, 1, 2, 2, 4, 5, and 10 minutes at 65°C. The bars correspond to the standard deviation of the mean (n = 4). **B**, Percentage of residual activity of papain in reactions with *Tc*CYS4 treated at 65°C and stored at 8°C (Control) over 20 days. The bars correspond to the standard deviations of the mean (n = 3). **C**, percentage of residual activity of papain in reactions with *Tc*CYS4 treated at 65°C, 80°C, and 65°C for 10 minutes and then treated at 80°C for 10 minutes, showing that the *Tc*CYS4 dimers were reconverted to monomers, when treated at 80°C. The activity of *Tc*CYS4-His-tag (histidine tailed) was similar to that of the protein *Tc*CYS4 (without histidine tail), demonstrating that the histidine tag does not interfere with the protein’s activity. The bars correspond to the standard deviation of the mean (n = 4). Means followed by the same capital letters, for the same inhibitor,do not differ among themselves by the Scott-Knott test at 5% probability. Means followed by the same lowercase letter, for the same treatment, do not differ among themselves by t test at 5% probability.

The time of exposure to the 65°C treatment influenced the inhibitory activity of *Tc*CYS4 (Fig. 3A). An increase in treatment time of *Tc*CYS4 at 65°C increased the residual activity of papain up to the time of 300 s. After this time of exposure, a plateau of residual activity of papain was formed, when has occurred an equilibrium between dimmers and monomers (Fig. 3A).

The treatment of *Tc*CYS4 at 65°C, followed by storage at 8°C over 20 days, indicated that the storage time did not alter the protein’s inhibitory activity against papain (Fig. 3B). When the oligomers formed at 65°C were treated at 80°C, the protein recovered the papain inhibition activity to a level similar to that prior to treatment at 65°C (Fig. 3C).

The presence of the His-tag did not affect significantly the inhibitory activity of *Tc*CYS4 in the samples without heat treatment and in those treated at 65, 80 and 65°C, followed by treatment at 80°C (Fig. 3C).

**Fig 4:**
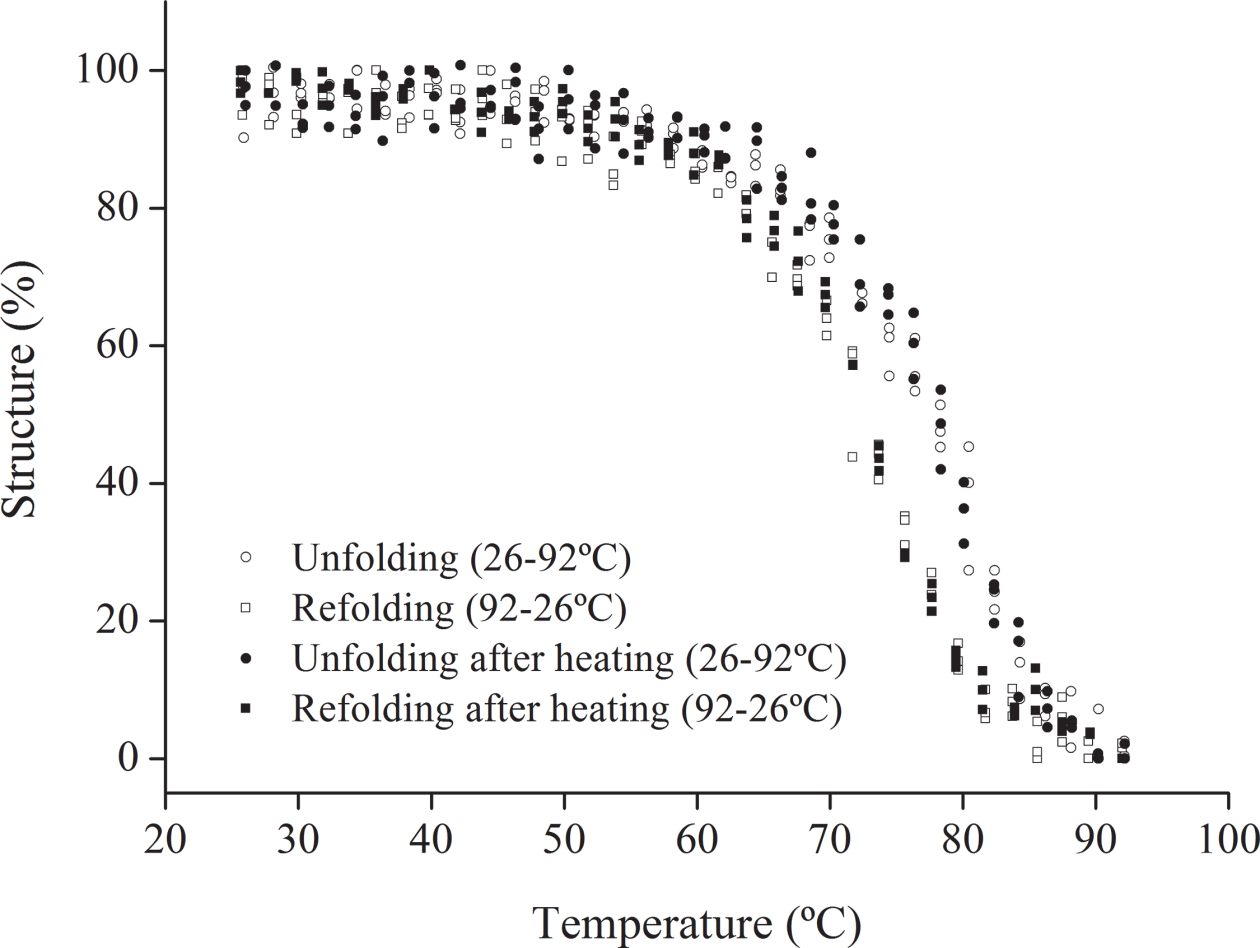
Unfolding and refolding of TcCYS4 expressed in percentage of structue. Unfolding of *Tc*CYS4 by heating from 26 to 92°C previously incubated at 8°C (empty circle) and 65°C (full circle), and refolding of this protein from 92 to 26°C previously incubated at 8°C (empty square) and 65°C (full square), showing that the protein pretreated at 65°C re-coils, similarly to protein pretreated at 8°C. Three spectra were performed to each treatment.”

The CD analyses showed that the heating of *Tc*CYS4 leads to a signal loss after 70°C (Fig. 4). In the analysis of unfolding and refolding by CD, it was observed that when the refolding of *Tc*CYS4 occurred, this protein did not return completely to its initial conformation, leading to a CD spectrum with a signal lower than the initial one, but with the same spectral profile (Fig. 4). Similar behavior was observed with the protein previously treated at 65°C; however, it was detected a lost of signal slightly greater for the protein previously treated at 65°C when compared with the protein without pre-treatment (Fig. 4).

### Influence from pH

**Fig 5:**
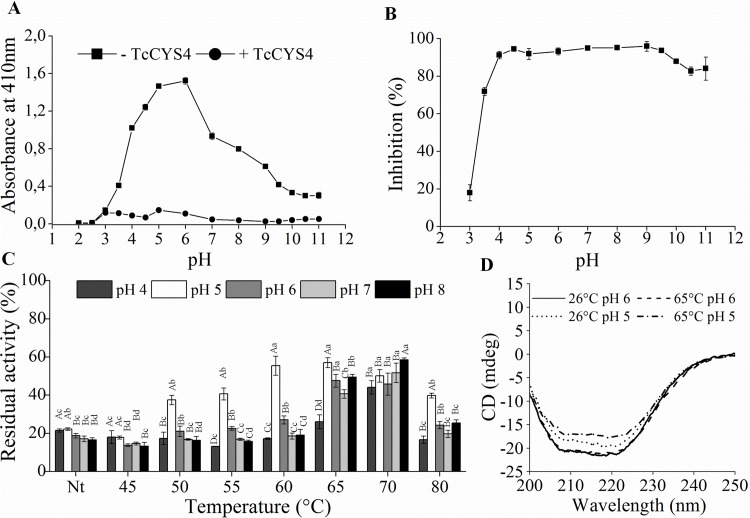
pH 5 induces the formation of *Tc*CYS4 dimers. **A**, Increases in absorbance of the reactions with *Tc*CYS4 (+*Tc*CYS4) and without *Tc*CYS4 (-*Tc*CYS4) at different pHs, showing that the inhibitory potential of *Tc*CYS4 was higher between pH 4 and 9. **B**, papain inhibition percentage of *Tc*CYS4 generated from A. The bars correspond to the standard error of the mean (n = 3). **C**, residual activity of papain in reactions containing *Tc*CYS4 under influence by the pHs 4, 5, 6, 7, and 8 associated with the heat treatment from 45 to 80°C. The bars correspond to the standard deviation of the mean (n = 3). Means followed by the same capital letters, for the same treatment of temperature, do not differ by the Scott-Knott test at 5% probability. Means followed by the same lowercase letters, for the same pH treatment, do not differ by the Scott-Knott test at 5% probability. **D**, profile of ellipticity by circular dichroism of *Tc*CYS4 at 26°C and pH 6 (solid line), 65°C and pH 6 (dashed line), 26°C and pH 5 (dotted line), and 65°C and pH 5 (dash and dot), showing that the treatments present similar profiles but the proteins at pH 5 present lower signals.

The effect of pH on the inhibitory activity of *Tc*CYS4 was analyzed (Fig. 5A). The values of absorbance for the reactions in the absence of the inhibitor have showed an optimum activity of papain around pH 6. In the presence of the inhibitor, it was observed a weak inhibition at pH values of 2 to 3, and above of pH 4 the inhibition increased (Fig. 5A). The percentage of inhibition for each pH value of the reaction medium was calculated (Fig. 5B). At pH 3 and 3.5, it was verified 20 and 70% of inhibition, respectively, while at pH 4 the inhibition of papain by *Tc*CYS4 was approximately 90%. This value was maintained up to pH 9, where the inhibition started to decrease, reaching approximately 80% at pH 10.5 (Fig. 5B).

*Tc*CYS4 was submitted to the different temperatures in combination with different pH values and the inhibitory activity was then evaluated in reaction medium at pH 6 (Fig. 5C). In the presence of *Tc*CYS4, without heat treatment and treated at 45°C at all the pH values analyzed, the residual activity of papain was of approximately 20%. In the treatment at 50°C in the pH 5, there was a remarkable increase in the residual activity of papain of ~40%, reaching ~60% at 65°C. The treatments at pH 6 also presented a slight increase in the residual activity of papain at the temperatures of 50, 55, and 60°C. In the treatment at 65°C, there was an increase in residual activity of papain in the pH range from 5 to 8. At this temperature, the residual activity of papain was of approximately 25, 60, 50, 40, and 50% for pH 4, 5, 6, 7, and 8, respectively. In the treatment at 70°C, pH 8 presented the greatest residual activity of papain, of ~60%, followed by the treatments at pH 5 and 7 with ~55%, and at pH 4 and 6 with ~50% of residual activity of papain. The treatment at 80°C was similar to the treatments at 50 and 55°C, at which pH 5 reached nearly 40% of residual activity of papain, followed by the treatments at pH 6 and 8, with ~25% and at pH 4 and 7 with ~20% of residual activity of papain (Fig. 5C).

The secondary structure of the *Tc*CYS4 was monitored by CD at pH 5 and 6 and temperatures of 26°C and 65°C (Fig. 5D). *Tc*CYS4, in the treatments at 26°C and 65°C at pH 6, presented similar ellipticity curves, both with peak of -22 mdeg (Fig. 5D). In the treatments under the influence of pH 5 at 26°C, the protein had a signal greater than at 65°C, with approximately -19 mdeg and -17 mdeg, respectively, however, presenting spectral pattern similar to that of the protein at pH 6.

### Acidity of the tissues of *T. cacao*

**Fig 6:**
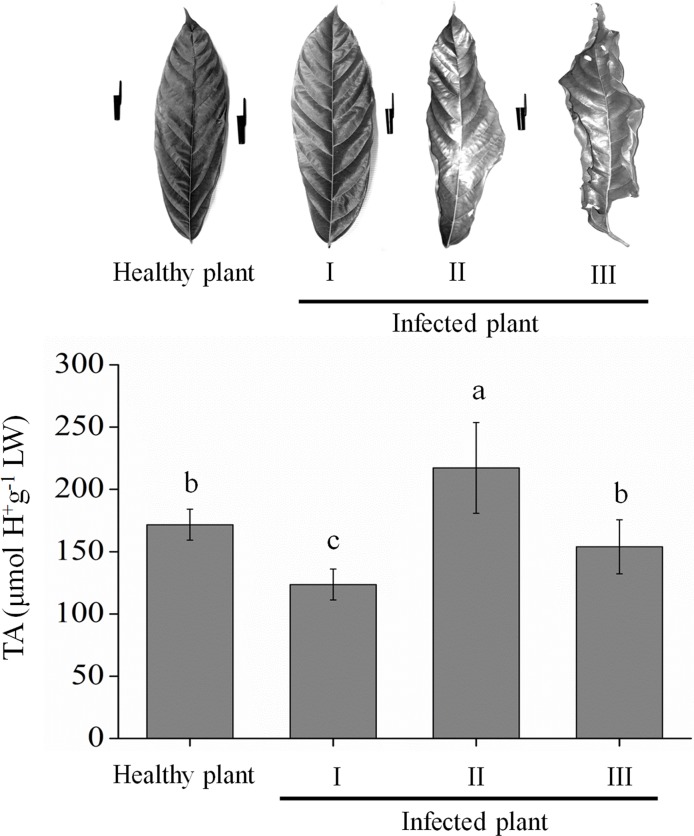
Variation of acidity in infected cacao leaves. **A**, image of the cocoa leaves with the same level of expansion and at each stage of development of the disease.**B**, Level of titratable acidity (TA) in healthy leaves and three developmental stages of witches’ broom disease. The bars correspond to the standard deviation of the mean (n = 5). Means followed by the same letters do not differ by the Scott-Knott test at 5% probability.

The titratable acidity (TA) was analyzed in leaves of healthy cocoa and in three stages of development of WBD symptoms (Fig. 6). The TA in leaves of healthy cocoa was of 170 μmol H^+^.g^-1^ FW (fresh weight). In leaves in the early stages of the parasitic phase of the disease (stage 1—first symptoms of hyperplasia and hypertrophy on the branch), the TA was of 120 μmol H^+^.g^-1^ FW. In the leaves of branches in late stages of the parasitic phase (stage 2—high degree of hyperplasia and hypertrophy on the branches and first signs of necrosis), the TA was the lowest, with 220 μmol H^+^.g^-1^ FW. In the leaves of branches at the beginning of the saprophytic phase of the disease (stage 3—branches partially necrotic and wrinkled leaves), the TA was of 150 μmol H^+^.g^-1^FW (Fig. 6).

## Discussion

### The extended C-terminal region may affect the thermostability of cystatins from *T. cacao*

Four cystatins were identified in ESTs libraries of interactions between cocoa and the fungus *M*. *perniciosa*, which causes WBD [11]. The heterologous proteins were produced in bacteria and decreased the growth rate of the saprophytic mycelium of the fungus *M*. *perniciosain vitro* [13]. In this study, we analyzed the effect of heat treatments and pH variation on the secondary structure and activity of these proteins named *Tc*CYS1, *Tc*CYS2, *Tc*CYS3, and *Tc*CYS4 [13]. The effects of temperature associated with pH variations on *Tc*CYS4 were also evaluated. *Tc*CYS1 is thermal unstable, since it presented loss of inhibitory activity against papain when it was submitted to an increase of temperature, while *Tc*CYS2, *Tc*CYS3, and *Tc*CYS4 are thermostable (Fig. 1). *Tc*CYS1 and *Tc*CYS3 present the signal peptide characteristic of proteins targeted to the secretory route and have sequence identity of 96% between them. *Tc*CYS3 is the short protein among them and it is originated from alternative splicing by exon skipping in the processing of the mRNA of *Tc*CYS1 [13], which results in a protein without the extended C-terminal region. This extended C-terminal region has a legumain-like protease inhibition domain [26] and it can be involved in the destabilization of the *Tc*CYS1 during heat treatment, once *Tc*CYS3 presents 96% of identity with *Tc*CYS1 and it is highly thermostable. The superposition of the curves of unfolding and refolding of *Tc*CYS3 (S1B Fig.) indicated that this protein recovers its native structure completely after treatment at 90°C.

The phytocystatins *Tc*CYS2 and *Tc*CYS4 have almost identical sequences at N-terminal and central regions; however, they presented 88% of identity due to the mutations that alter the C-terminal region of the protein. Only 11 C-terminal residues are differentes. A premature stop codon was observed in *Tc*CYS2, making it smaller than *Tc*CYS4, which contains an extended C-terminal region [13]. *Tc*CYS2 and *Tc*CYS4 showed thermostability when submitted to different temperatures, however *Tc*CYS2 is more affected by high temperatures than *Tc*CYS4 (Fig. 1), and both showed the lowest inhibitory activity when they were treated at 65°C. The presence of the extended C-terminal tail in *Tc*CYS4 may have made this protein more stable than the protein *Tc*CYS2 when expressed in *E*. *coli*. During heterologous expression, the protein *Tc*CYS2 was accumulated in the insoluble fraction of the bacterial extract, while *Tc*CYS4 accumulated in the soluble fraction. This suggests that the presence of the extended carboxy region may assist in the stabilization and folding of the protein expressed in *E*. *coli* [13].

Although TcCYS1 have extended C-terminal tail, similarly to TcCYS4, they have only 46% sequence identity [13] and showed opposite behavior in terms of thermostability (Fig. 1).

### The exchange domain may be responsible for the formation of *Tc*CYS4 dimers

*Tc*CYS2 and *Tc*CYS4 are thermostable, but presented an intriguing behavior with a peak of activity reduction when treated at 65°C (Fig. 1). *Tc*CYS4 is abundantly obtained from the soluble fraction of the bacterial extract, while *Tc*CYS2 is only recovered from the insoluble fraction by solubilization with urea and recovered in the active soluble form at low concentration [13]. For this reason, only *Tc*CYS4 was used in the analyses of oligomerization and inhibition. In the analysis of oligomerization of the *Tc*CYS4 through native gel electrophoresis (Fig. 2A) and size-exclusion chromatography (Fig. 2C), the presences of oligomers and monomers were observed in all the heat treatments and in the control sample, with predominance of the monomeric form in comparison with the oligomeric form (Fig. 2A, B, and C). Thus, it is likely that dimers and oligomers exist naturally in the protein; however, the concentration ratio of the two forms may vary depending on the conditions of the solution for the recombinant protein or on the cellular condition, for the endogenous *T*. *cacao* protein. The analyses indicated higher proportion of dimers and trimers in the treatment at 65°C, and the protein’s inhibitory activity against the target protease was proportional to the quantity of dimers. This becomes evident when the curves of residual activity of papain (Fig. 1) and of relative quantity of monomers (Fig. 2B) are compared. Thus, the oligomers can be considered an inactive form of the *Tc*CYS4, and the interconversion between oligomers (inactive form) and monomers (active form) may constitute a mechanism of regulation of the activity of the endogenous protein. The oligomerization may be caused by an extension at the C-terminal region, which would allow the recognition and binding of conserved motifs, with the participation of the N-terminal region [27].

It was verified that the human cystatin C has the capacity to form dimers [28], similarly to *Tc*CYS4. The human Cystatin C is functional in its native monomer state, and the presence of inactive dimeric forms in pathological conditions is related to the presence of an “exchange domain” that leads to the dimerization of the protein from its monomers [29,30]. The structure of the cystatins consists of a framework of five β-sheets with one central α-helix [31]. The “exchange domain” is the exchange of an α-helix of a protein for the α-helix of the other, forming a stable structure due to new interactions, such as the one that occurs between the amino acids Ile 56—Gly 59 of the different monomers; and this cohesive structure is responsible for the stability of the dimers that are formed [31].

Recently, the formation of dimers through exchange domain was described for canecystatin 1 of *Saccharum officinarum*. This was the first report on the formation of dimers of plant cystatin from this domain [32]. This protein has greater flexibility in the N-terminal region, which may produce a twist in the central portion of the β-sheet that is greater than that described for human cystatin C [32]. Thus, it is possible that similar structures can be found in *Tc*CYS4, providing the protein with the capacity of forming homodimers and homotrimers, as observed in Fig. 2C, through the cohesive interaction between the amino acids of adjacent molecules. This can lead to the formation of stable oligomers of *Tc*CYS4.

In the analysis of *Tc*CYS4 through CD, no significant conformational alterations occurred in the treatments at 20 and 65°C, as revealed by the spectral profiles of the protein (Fig. 2D). This suggests that the dimers are possibly formed by exchange domain, because the CD spectra indicate that no alteration occurs in the proportion between α-helices and β-sheets. The analysis of the formation of dimers and tetramers in human cystatin C through CD also showed that there were no conformational alterations in this protein [33]. This is due to the fact that, during the formation of the dimers of cystatin C, the exchange of one α-helix of a molecule for the α-helix from the other occurs without alteration in the proportion of secondary structures of the protein. Moreover, in the model of formation of canecystatin dimers proposed in [32], in order to form dimers and tetramers, the structures of the monomers share among themselves one β-sheet, one α-helix, and one loop, causing the connection of the structures; however, there was no alteration in the content of regular secondary structures. This model could be applied to the dimerization mechanism of *Tc*CYS4, a fact that would justify the small variation in the CD signals in the treatments at 20 and 65°C.

Concerning the treatment at 90°C in comparison with the treatments at 20 and 65°C, alteration in the spectral profile of the protein was observed, showing that the temperature of 90°C promotes the loss of the regular secondary structure of the TcCYS4.

### The oligomerization of *Tc*CYS4 is a fast and endergonic process, and the oligomers formed are stable and require a great amount of energy to be reconverted to monomer

The papain inhibition assay with *Tc*CYS4 treated at 65°C for time intervals from 0 to 600 s indicates that the oligomerization of the protein occurs in approximately five minutes (Fig. 3A) under the conditions of our study.

The residual activity of papain started at approximately 20% and reached near 60% with the increase in the time of heat treatment of the cystatin. We emphasize that only a fraction of the protein is oligomerized (Fig. 2A, 2B, and 2C). The concentration of the protein and the temperature are factors that directly influence the oligomerization [34]. The temperature around 65°C provides the energy required for the conversion of monomers of *Tc*CYS4 into dimers (Fig. 2A, 2B, and 2C). The increase of the proportion of dimers with the increase of treatment time can be explained by the fact that the higher the treatment time, the longer the sample will receive energy for the conversion of monomers to dimers. Moreover, as the dimers are formed, a reduced amount of free monomers will be available to be converted to the oligomeric form, due to the reduction of the probability of contact between monomer molecules in the reaction medium. We also hypothesized that, *in vivo*, the removal of oligomers from the medium by precipitation or interaction with molecular chaperones may favor the complete oligomerization of the protein. Moreover, our assays showed that the oligomers formed at 65°C are stable, since the papain inhibitory activity of *Tc*CYS4 underwent small alteration after treatment at 65°C and storage over 20 days at 8°C (Fig. 3B). This indicates that the dimers formed during the heat treatment are stable during storage. In the evaluation of the reconversion of the dimers to monomers (Fig. 3C), it was observed that the recombinant *Tc*CYS4 treated at 65°C and then at 80°C presented activity similar to the protein treated only at 80°C. This suggests that the oligomers formed at 65°C were reconverted to active monomers. This explains why the inhibitory potential of *Tc*CYS4 when treated at 80°C is higher than that of the protein without heat treatment (Fig. 1), since the dimers (inactive form of *Tc*CYS4), which exist naturally in the sample at 8°C, were converted to monomers (active form of the protein) in the treatment at 80°C. Similar assays with recombinant oryzacystatin-II [22] showed a behavior opposite to that of *Tc*CYS4. The protein of rice was purified from the bacterial extract with a higher proportion of inactive dimers, and a higher proportion of active monomers was formed at 65°C; however, they were rapidly reconverted to dimers when stored at 4°C. In this case, the active monomeric form of the rice cystatin and the inactive oligomeric form of the cocoa cystatin depend on energy to be formed. During the interactions in the exchange domain of the human cystatin C, there is a great energy barrier separating the monomeric and dimeric forms [34]. This indicates that there is a great energy cost to break and replace the interactions of the exchange region, which typically represent a major portion of the total of contacts in the monomer. Moreover, the kinetic accessibility of the dimers formed through exchange domain significantly decreases when there is competition between the monomeric and dimeric forms, showing that the exchange between the two forms requires crossing a high energy barrier [34].

Another factor influencing the conversion of dimers to monomers is the fact that, once the heat treatment is established, in which energy is supplied to the sample, the proteins slightly lose their conformation, becoming “more open” and more flexible. This fact makes possible the formation of dimers by exchange of domains between these more distended molecules. If more energy is supplied to the system containing these proteins in the form of dimers, it is possible that these dimers will tend to distend again, due to the breaking of the connections established, and will separate by the increase of the entropy of the system, in order to promote the dissociation of the dimers to monomers [34].

Based on the knowledge that the dimers of *Tc*CYS4 are reconverted to monomers when treated at 80°C for 10 minutes, whether oligomerization occurs as a form of regulation of *Tc*CYS4 *in vivo*, this physiological process most likely will be irreversible, considering the high energy demand for the reconversion to monomers *in vitro*. Thus, this regulation process must be more appropriate in cellular processes that are also irreversible, such as programmed cell death (PCD) or cell necrosis, which occurs in the tissues of *T*. *cacao*, during the shift from the biotrophic to the parasitic phase of *M*. *perniciosa* [5]. It has been proposed that the balance between proteases and their protein inhibitors may be crucial to trigger PCD and they may be involved in the molecular battle involving the interaction between plant and pathogen [35].

The curves of the unfolding and refolding of the protein previously treated at 65°C are similar to those of the untreated protein (Fig. 4). This result indicates that the pre-treatment at 65°C, may induce oligomerization, but do not affect stability of the secondary structure from protein.

### pH may affect the oligomerization of *Tc*CYS4

*Tc*CYS4 presented capacity of inhibiting papain in a wide range of pH (4–11). The inhibition was only reduced at pH below 3.5 (Fig. 5A and B). Possibly, the extreme pHs influence the native conformation of the protein, so that, in these pHs, reduction of the capacity of interacting with the catalytic cleft of the papain occurs. On the other hand, the acidic pH may also affect the structure of papain. In this sense, the area of contact between the cystatin and the target proteases, in the models of docking, involves hydrophobic and ionic interactions that may be affected by variations in pH [36]. In addition, many proteins do not support great variations in pH, with denaturation occurring at extreme pH ranges; however, *T*cCYS4 supported a wide range of variation of pH without losing its inhibitory power completely, proving to be more sensitive to extremely acidic pHs.

We analyzed the effect of heat treatment on the *Tc*CYS4 in the pH range of 4–8 (Fig. 5C). The treatments from 50°C at pH 5 presented residual activity of papain higher than in the other pH values. This suggests that the oligomerization of the protein during heating may be favored at pH 5. The CD spectra of the protein treated at 26°C and 65°C at pH 6, compared with the treatment at pH 5 (Fig. 5D), presented similar curves, but with a slight reduction of signal for the treatments at pH 5. This suggests that the structure of *Tc*CYS4 may be slightly more flexible and promote oligomerization at pH 5. Another factor that may affect the CD signal of the protein treated at 65°C is the less significant exposure of the chiral center carbons of the oligomers in comparison with the monomers. In the oligomers, the chiral center carbons may be less exposed to the plane-polarized light, and this can result in a less intense signal for the treatments that favor oligomerization.

At pH 5, there was a marked reduction in the inhibitory activity of *Tc*CYS4 in almost all the heat treatments evaluated (Fig. 5C). The titratable acidity (TA) analyses in cocoa leaves infected by *M*. *perniciosa* showed a rise of pH at the beginning of the saprophytic phase and at the beginning of the necrotrophic phase of WBD (Fig. 6). The increased inactivation of *Tc*CYS4 at pH 5, in mild temperatures (50°C), observed *in vitro*, also suggests that, *in vivo*, the dimerization at pH 5, although still endergonic, presents less dependence on energy than at other pH values. However, the connection of energy producing systems in the cell to this oligomerization remains to be demonstrated.

During the *M*. *perniciosa* infection in *T*. *cacao*, there is an increase in the content of salicylic acid [37]. In other species, this phenomenon is responsible for increasing the temperature of the plant’s tissues in up to 14°C [37,38]. Another factor that may also contribute to the increase of temperature during the interaction between *T*. *cacao* and *M*. *perniciosa* is the uncontrolled respiratory processes and the loss of water of the tissues in the later stages of infection [25]. This can lead to the reduction of the relative water content in the tissues and hence to adiabatic heat loss, reducing the thermal tamponade. In the advanced stages of WBD, the cacao tree leaves become dry and shriveled [25] (Fig. 6). In these conditions, the leaves may suffer temperature increase due to the incapacity of regulating transpiration. An important effect of CO_2_ increase is the increased acidification of the tissues caused by the dissociation of the carbonic acid into bicarbonate and hydrogen ions, and the increase of the CO_2_ content in 5% is capable of causing reductions in the intracellular pH [39]. The cytoplasmic and vacuolar pH was evaluated through magnetic nuclear resonance in tissues of *Lactuca sativa*, exposed to air at 20°C and stored for 6 days at 0°C with 16% of CO_2_, and it was found that the pH decreased in 0.4 and 0.1 units in the cytoplasm and vacuole, respectively [40]. In advanced stages of the infection by *M*. *perniciosa*, a type of respiratory upsurge may occur [5] and lead to increase of CO_2_ in the tissues, causing increased acidity of the leaf tissues of *T*. *cacao*. The increase of the temperature of the plant tissues can also lead to an increased acidity due to the increased levels of CO_2_ [39]. During the process of cell death, leakage of vacuolar contents or contents from other organelles can also occur [5], which may acidify the cytoplasm of the cells in advanced stages of infection. The dynamics of synthesis and dissolution of calcium oxalate drusen during the disease’s parasitic phase [5,9] may also favor the variation of pH in the interaction between cocoa and *M*. *perniciosa*. Additionally, NEP1 (necrosis- and ethylene-inducing peptide 1) produced during changing of the parasitic to the necrotrophic phase of the witches broom, when introduced in cell suspension culture of the *Nicotiana benthamiana*, induces a rapid proteome and metabolome reprogramming, causing a change in the plant cell metabolism, from aerobic respiration to anaerobic fermentation [41]. This can acidify the citosol and promote the inactivation of TcCYS4. Therefore, the increase in the intracellular concentration of *Tc*CYS4 in young tissues and during the parasitic phase of the disease, associated with a increase of temperature, resulting from the increased levels of salicylic acid, and from the decrease of pH, due to the leakage of vacuolar contents and increase of CO_2_, and the reduction of the relative water content in the tissues. This may increase the interactions between the monomers, may be associated with the displacement in the Keq for the formation of *Tc*CYS4 dimers, thus overcoming the energy barrier of this process.

In conclusion, the heterologous cystatin *Tc*CYS4 can be found in the monomeric or oligomeric forms depending on the conditions of pH and temperature, but at 65°C and pH 5 the protein reduces inhibitory activity. *Tc*CYS4 dimers are stable, but can be reconverted to monomers after another treatment at 80°C. In a living system, cystatins perform various functions, including the protection of tissues against pathogens. The presence of oligomeric forms with distinct inhibitory potentials *in vitro* suggests that oligomerization *in vivo* may regulate the protein’s activity during the symptoms of WBD, caused by the fungus *M*. *perniciosa* in cocoa. Thus, in the initial stages of the disease, the protein *Tc*CYS4 in its active form can inhibit cysteine proteases and prevent cell death, and also to inhibit proteases of the digestive tract of herbivores. As *M*. *perniciosa* establishes and manages to overcome the initial defense barriers of *T*. *cacao*, *Tc*CYS4 can be converted to a less active form (oligomers) through stimuli resulting from the plant’s physiological responses to the pathogen attack (stomatal closure; respiratory upsurge; possible temperature rise due to increase of jasmonic acid, salicylic acid, and CO2; and chaperone activity). This makes possible that the previously inhibited cysteine proteases act on the programmed cell death, causing death to the tissues in the final stages of the disease. This is in accordance with the balance between proteases and their inhibitors in the regulation in the molecular battle between plant and pathogen [35].

## Supporting Information

S1 Fig The *Tc*CYS3 protein restructured after heat treatment. **A,** CD spectra of TcCYS3 at concentration of 0.25 mg mL^1^ in 10 mM sodium phosphate, pH 7.2, at 25°C. **B**, Unfolding of *Tc*CYS3 by heating from 20 to 95°C (black ball), and refolding of this protein from 95 to 20°C (red triangle), showing that protein re-coils upon heating to 95°C because of the overlap of the unfolding and refolding occurs profiles showing no loss of signal or structure.
(TIF)
