# Supplementary figures and images for: The Activity of TcCYS4 Modified by Variations in pH and Temperature Can Affect Symptoms of Witches’ Broom Disease of Cocoa, Caused by the Fungus Moniliophthora perniciosa
Source: PLoS One. 2015 Apr 1;10(4):e0121519. doi: 10.1371/journal.pone.0121519 (PMC4382335; doi:10.1371/journal.pone.0121519)

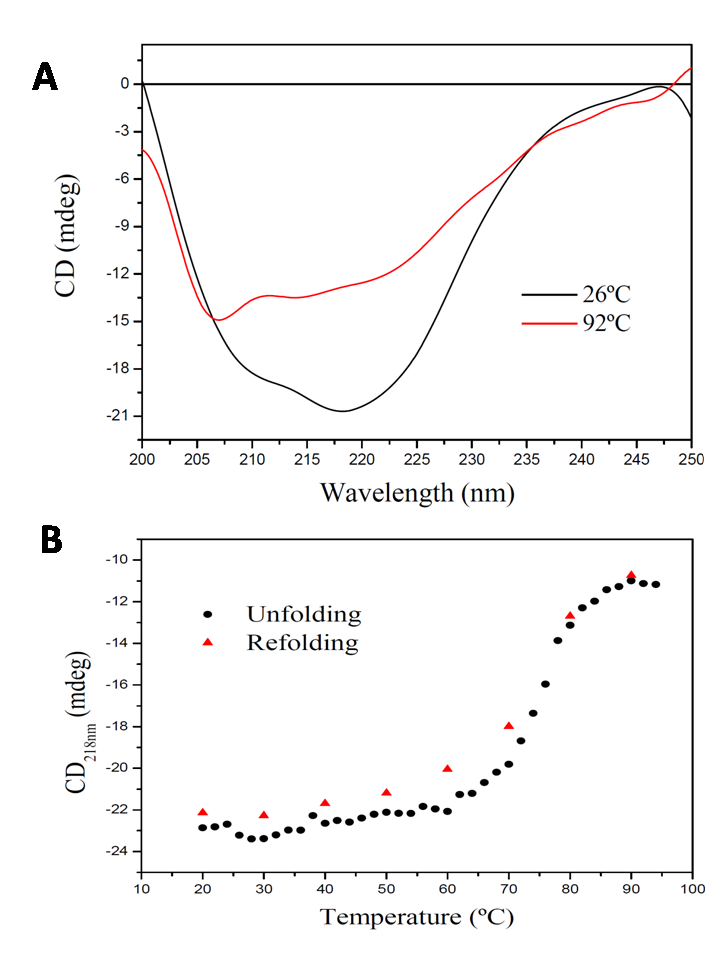

Supplement: S1 Fig — A, CD spectra of TcCYS3 at concentration of 0.25 mg mL1 in 10 mM sodium phosphate, pH 7.2, at 25°C. B, Unfolding of TcCYS3 by heating from 20 to 95°C (black ball), and refolding of this protein from 95 to 20°C (red triangle), showing that protein re-coils upon heating to 95°C because of the overlap of the unfolding and refolding occurs profiles showing no loss of signal or structure. (TIF) [file pone.0121519.s001.tif]
